# Being a Partner or a Mother: Breastfeeding Mothers’ Perceptions and Experiences of Breast Milk and Sexuality in the Postpartum Period

**DOI:** 10.1007/s10508-026-03540-z

**Published:** 2026-10-05

**Authors:** Pınar Akbaş, Sultan Özkan Şat

**Affiliations:** 1 Nursing Department, Faculty of Health Sciences, Karabük University, Karabük, Turkey; 2 Midwifery Department, Faculty of Health Sciences, Bitlis Eren University, Bitlis, 13100 Turkey

**Keywords:** Breast milk, Sexuality, Breastfeeding, Postpartum period

## Abstract

This study aimed to explore breastfeeding mothers’ perceptions and experiences related to breast milk and sexuality between the third and sixth months postpartum. A descriptive qualitative design was employed. The sample consisted of 10 mothers, including primiparous (*n* = 4) and multiparous (*n* = 6) women, with a mean age of 29 years. Data were collected through individual semi-structured interviews. Thematic analysis was used to analyze the data. Three main themes emerged from the analysis: avoidance of sexuality, maintaining sexuality as a responsibility, and coping with the effects of breastfeeding on sexuality. Six subthemes were identified within these main themes: physical changes in sexual dynamics; psychological changes in sexual dynamics; religious, social, and cultural influences on sexuality; fulfillment of the female role; changing perceptions of breasts; and various coping strategies. The findings suggest that breastfeeding and postpartum physiological and sociocultural factors shape mothers’ sexual experiences, with sexuality often being perceived as inappropriate during this period due to cultural and religious norms, while participants’ perceptions related to breast milk also contribute to these experiences. Mothers attempt to balance breastfeeding with maintaining their sexual relationships, which frequently leads to emotional distress. It is recommended that healthcare professionals address breastfeeding, breast milk, and sexuality within breastfeeding education programs and provide individualized, culturally sensitive guidance for couples.

## Introduction

Sexuality is a lifelong phenomenon that is perceived and experienced differently across various stages of an individual’s life (O’Malley et al., 2019). In this context, the postpartum period represents a critical phase during which women adapt to the maternal role while undergoing substantial physical, psychological, social, and cultural changes (Ollivier et al., 2020). Numerous factors, including hormonal fluctuations, breastfeeding, infant care responsibilities, fatigue, changes in body image, and adaptation to new social roles, may influence women’s sexual health and perceptions of sexuality during this period (Khajehei & Doherty, 2018).

Women typically resume sexual activity between 6 and 8 weeks following childbirth (McDonald et al., 2017). However, the physical and psychological adjustments characteristic of the postpartum period may adversely affect sexuality, potentially leading to challenges within intimate partner relationships (Khajehei & Doherty, 2018). Among the factors influencing postpartum sexuality, breastfeeding has been identified as one of the most significant (Szöllősi & Szabó, 2021). Although breastfeeding is a physiological process that promotes bonding between mother and infant and may provide pleasure for both, it can complicate the maintenance of sexual relationships between partners and contribute to disruptions in sexual life (Delgado-Pérez et al., 2022). A recent meta-analysis assessing sexual dysfunction among breastfeeding women showed that, regardless of feeding method, exclusive breastfeeding, mixed feeding, or formula feeding, many women experienced sexual difficulties during the postpartum period. Notably, higher rates of sexual dysfunction were observed among women who exclusively breastfed (Smetanina et al., 2025). These findings suggest that breastfeeding is a significant factor that may negatively influence sexuality during the postpartum period.

The relationship between breastfeeding and sexuality has predominantly been examined through a physiological lens (Gutzeit et al., 2020; O’Malley et al., 2018; Szöllősi & Szabó, 2021). From a physiological perspective, breastfeeding is associated with hormonal changes that may influence sexual functioning in the postpartum period. Elevated prolactin levels, which support lactation, are commonly linked to suppressed estrogen production. This hormonal pattern may contribute to reduced vaginal lubrication, decreased sexual desire, and changes in sexual responsiveness among breastfeeding women (Adeli et al., 2026; Jeong et al., 2021). These physiological changes provide an important context for understanding how breastfeeding may shape women’s sexual experiences.

Postpartum sexuality is shaped not only by physiological changes but also by a range of psychological, social, and cultural factors. Psychologically, women may experience changes in body image, fatigue, sleep disruption, and postpartum mood fluctuations, all of which can influence sexual desire and sexual self-perception (Gmelig Meyling et al., 2023). Socially, relationship dynamics, partner support, division of childcare responsibilities, and perceived expectations regarding motherhood may play an important role in shaping sexual adjustment during the postpartum period (Arampatzi et al., 2025; Hipp et al., 2012). Culturally, beliefs surrounding motherhood and female sexuality can strongly influence how women interpret sexual activity after childbirth. In some contexts, sexual activity during the postpartum period may be viewed as secondary to maternal responsibilities, which can affect communication about sexual needs and experiences (Ollivier et al., 2024; Rahmani et al., 2023). In addition, religion may represent an important contextual factor shaping postpartum sexuality. In some societies, breast milk is given spiritual significance, and breastfeeding is considered sacred, with religious meanings attributed to it. This is reported to negatively affect sexuality during breastfeeding and lead to avoidance of sexual activity (Cantarino et al., 2016; Hirani & Ratushniak, 2022). This is particularly relevant in settings where religious norms are closely intertwined with cultural expectations of motherhood, such as Türkiye.

Given the complexity of this phenomenon, the impact of breastfeeding on sexuality cannot be adequately explained solely by physiological changes. Rather, it necessitates a comprehensive examination that incorporates psychological, social, and cultural perspectives (Hipp et al., 2012; Martínez-Martínez et al., 2017). Although it has been reported that factors such as a woman’s feeling of being desired by her partner, body image, social support, and spirituality can have an impact on sexual health during breastfeeding, the psychological, social, and cultural dimensions of the relationship between breastfeeding and sexuality in the postpartum period have not been sufficiently researched (Hipp et al., 2012). In line with this perspective, there remains a notable gap in the literature regarding context-specific, in-depth explorations of women’s lived experiences. To our knowledge, this is the first qualitative study conducted in Türkiye that explores the sexual experiences of breastfeeding women. While previous studies have largely focused on biological and physiological aspects of postpartum sexuality, limited attention has been given to how sexuality is shaped within specific cultural and social contexts.

In Türkiye, cultural norms and religious values may strongly influence perceptions of motherhood, breastfeeding, and sexuality (Celebier et al., 2025). These sociocultural frameworks may shape how women experience and interpret their sexual lives during the breastfeeding period, highlighting the need for context-sensitive research that goes beyond purely biological explanations. It is also important to recognize that postpartum sexual experiences may differ between birthing and non-birthing partners. Birthing women experience direct physical recovery and hormonal changes, while non-birthing partners mainly experience relational and emotional changes in the couple relationship (Ahlborg et al., 2005). In addition, non-birthing lactating parents, such as those who experience induced lactation, may face sexual and psychosocial challenges related to hormonal changes, bodily adaptation, and breastfeeding-related demands (Goldberg, 2010). This distinction is particularly relevant in diverse family structures, including same-sex couples, where birthing and lactating roles may not necessarily overlap (Patterson, 2022). This underscores the importance of clearly defining the conceptual and empirical scope of the study population. Based on this context, this study focuses on cisgender heterosexual women in the postpartum period in Türkiye. This is a deliberate methodological choice, as this group represents the most commonly studied population in the literature on breastfeeding and postpartum sexuality. The aim is not to suggest exclusivity or greater relevance of this group, but to define the scope of the study and allow for an in-depth exploration of breastfeeding women’s sexual experiences within a specific sociocultural context. Accordingly, this study aims to conduct an in-depth exploration of breastfeeding women’s perceptions and experiences regarding breast milk and sexuality in the postpartum period. It is anticipated that the findings will illuminate underexplored aspects of the relationship between breastfeeding and sexuality and contribute a novel perspective to the existing literature.

## Method

### Participants

The study was conducted with breastfeeding women who were between the third and sixth months postpartum and were receiving follow-up care at a family health center in a province in northern Türkiye. The province is a socioculturally cosmopolitan and highly industrialized region, encompassing individuals with diverse cultural backgrounds from across Türkiye.

A purposive sampling strategy was used to recruit participants who met predefined inclusion criteria relevant to the aims of the study. This approach ensured that individuals with specific characteristics and experiences were intentionally selected in order to obtain rich and relevant data regarding breastfeeding mothers’ experiences related to breast milk and sexuality. Inclusion criteria were being aged 18 years or older, being between the third and sixth months postpartum, and being actively breastfeeding. The 3–6-month postpartum period was selected for this study because it generally corresponds to the phase in which sexual activity is commonly resumed, while breastfeeding remains highly prevalent in Türkiye, as reported by the Türkiye Population and Health Survey (Hacettepe University Institute of Population Studies, 2019). This timeframe was therefore considered appropriate to capture the overlap between ongoing breastfeeding and the re-emergence of sexual activity. Although sexual activity may resume as early as 6–8 weeks postpartum according to clinical recommendations and existing literature, the aim of this study was to explore women’s lived experiences after the initial postpartum recovery period, when sexual activity and breastfeeding frequently coexist. Selecting this period allowed for an in-depth examination of postpartum sexuality within the context of active breastfeeding, providing a more comprehensive understanding of this transitional phase. Women with communication impairments (e.g., inability to speak or understand Turkish, visual, hearing, or speech impairments, or disorganized behavior related to psychiatric diagnoses) and those living separately (i.e., not currently cohabiting with a spouse or partner) were excluded from the study. Therefore, participants were recruited as postpartum individuals who self-identified as women and were currently breastfeeding. Accordingly, the sample consisted of postpartum women living with a spouse or partner within the Turkish cultural context.

In qualitative research, sample size is determined based on the principle of data saturation rather than statistical representativeness. Data collection is continued until no new themes or meaningful insights emerge from additional interviews (Morse, 2015). In the present study, interviews were conducted until data saturation was achieved, as similar responses and recurring themes were observed and no new themes emerged in the final interviews.

Furthermore, the literature indicates that sample size in qualitative studies depends on the scope of the research question and the homogeneity of the sample. When the research question is narrow in scope and the sample is relatively homogeneous, smaller sample sizes may be sufficient, whereas broader and more complex research questions with heterogeneous samples may require larger sample sizes (Dworkin, 2024). In this study, the sample was relatively homogeneous, consisting of breastfeeding mothers in the postpartum period between 3 and 6 months who shared similar physiological and experiential characteristics. Therefore, considering the focused nature of the research question and the homogeneity of the study group, a sample size of 10 participants was deemed adequate. Although the sample included both primiparous and multiparous women, no substantial differences were observed in terms of emerging themes during data analysis, supporting the adequacy of the sample size.

A descriptive qualitative research design was employed to explore participants’ thoughts, perceptions, and experiences in depth (Creswell & Creswell, 2014). This approach was considered appropriate to gain insight into breastfeeding mothers’ experiences regarding breast milk and sexuality and to identify their perceived needs. The study was further guided by a thematic analysis framework, which informed both data collection and analysis. Accordingly, the semi-structured interview guide was developed in line with the research aims and refined iteratively during the data collection process. Data collection and analysis were conducted concurrently, and recruitment continued until data saturation was reached, defined as the point at which no new themes emerged. The study was reported in accordance with the Consolidated Criteria for Reporting Qualitative Research (COREQ) checklist (Tong et al., 2007).

### Measures and Procedure

Data were collected using individual, in-depth interviews. Prior to the interviews, all participants were informed about the purpose of the study, and written informed consent was obtained. Participants also completed a personal information form. The personal information form included sociodemographic and contextual variables such as age, education level, employment status, obstetric characteristics (e.g., number of pregnancies and births), and postpartum status. In addition, participants were asked about their marital or relationship status (being married or living with a partner) and religious affiliation, considering the sociocultural context of Türkiye where the study was conducted.

To ensure the content validity of the semi-structured interview form, expert opinions were obtained. The draft form was evaluated by six experts with relevant field experience, including three experts in Obstetrics and Gynecology Nursing and three experts in Midwifery. Each item was assessed using a four-point rating scale (1 = not relevant, 2 = needs major revision, 3 = relevant but requires minor revision, 4 = highly relevant) in accordance with the Davis technique. The Content Validity Index (CVI) was calculated based on the proportion of experts who rated each item as either 3 or 4. For the relevant item, five out of six experts provided ratings of 3 or 4, yielding a CVI value of 0.83, which indicates an acceptable level of content validity (Davis, 1992). In line with expert feedback, necessary revisions were made to the interview questions.

A semi-structured interview guide was used, and probing questions were asked when necessary to deepen participants’ responses. To assess participants’ awareness of breastfeeding and sexuality and to facilitate engagement, the interviews began with an icebreaker question: “It can be difficult to resume sexual intercourse after childbirth. What would you like to say about this?” The semi-structured interview guide is presented in the Appendix.

Each participant took part in a single interview session. Interviews were conducted in accordance with confidentiality principles and were audio-recorded with participants’ permission. In addition, field notes were taken to support data interpretation. The interviews lasted approximately 30–40 min and were conducted in a private and comfortable room within the health center, free from potential distractions that could affect the interview process.

### Data Analysis

Data were analyzed using the thematic analysis framework proposed by Braun and Clarke (2006). This approach involves six sequential phases: familiarization with the data, generation of initial codes, searching for themes, reviewing themes, defining and naming themes, and producing the final report. The study findings were systematically organized and presented under overarching themes, supported by illustrative excerpts from the interview data to enhance transparency and depth of interpretation.

### Rigor

The trustworthiness of the study was ensured in accordance with the criteria of credibility, transferability, dependability, and confirmability.

Credibility: Braun and Clarke’s (2006) thematic analysis method guided the analytical process. Themes derived from participants’ narratives were substantiated with direct quotations to ensure that the findings accurately reflected participants’ lived experiences. In addition, selected themes were shared with participants for member checking, and their feedback was incorporated to confirm interpretive accuracy.

Transferability: To facilitate the evaluation of the applicability of the findings to similar contexts, detailed descriptions of participants’ sociodemographic and obstetric characteristics were provided. Furthermore, the research context, including the setting, timing, and environment of the study, was clearly delineated.

Dependability: The procedures for data collection, interview conduct, and data analysis were described in detail. All interview transcripts were independently reviewed and coded by two members of the research team. Following independent coding, the researchers compared their codes and preliminary themes, and any discrepancies were discussed in detail. Consensus was reached through iterative discussion and re-examination of the data until agreement was achieved. In cases where differences persisted, an independent expert in midwifery was consulted to facilitate resolution. This collaborative and iterative process contributed to enhancing the credibility and dependability of the analysis.

Confirmability: Reflexivity was continuously applied throughout the research process to minimize the influence of researchers’ assumptions and biases. The research team’s interpretations were consistently grounded in participants’ statements rather than pre-existing expectations. As part of this process, coding and theme development were cross-checked among researchers through iterative discussion and comparison of interpretations. The researchers’ professional backgrounds in maternal and reproductive health were acknowledged and critically reflected upon throughout data collection and analysis to reduce potential interpretive bias. Objectivity and analytic rigor were further strengthened through consultation with an independent expert in midwifery who was not involved in the study.

Through these strategies, care was taken to enhance the credibility, dependability, and trustworthiness of the findings.

## Results

Participants’ characteristics are presented in Table 1. The study sample consisted of 10 mothers, including primiparous (*n* = 4) and multiparous (*n* = 6) women, with a mean age of 29 years. Participants reported resuming sexual intercourse as early as the second month and as late as the sixth month postpartum (Table 1).

**Table 1:** Sociodemographic and obstetric characteristics of the participants

ID Code	Age	Education	Employment	Total number of pregnancies	Time to start having sex after childbirth	Religious affiliation
1	33	High school	No	3	Third month	Islam
2	28	High school	No	2	Fifth month	Islam
3	25	University	Yes	1	Sixth month	Islam
4	22	University	No	1	Fourth month	Islam
5	38	High school	No	4	Fourth month	Islam
6	31	High school	No	1	Third month	Islam
7	21	High school	No	1	Fourth month	Islam
8	32	University	Yes	2	Second month	Islam
9	34	High school	No	2	Third month	Islam
10	26	High school	Yes	3	Second month	Islam

Three main themes were identified in this study, which explored breastfeeding mothers’ perceptions and experiences related to breast milk and sexuality between the third and sixth months postpartum: avoidance of sexuality, maintaining sexuality as a responsibility, and coping with the effects of breastfeeding on sexuality. These overarching themes comprised six subthemes: physical changes in sexual dynamics; psychological changes in sexual dynamics; religious, social, and cultural influences on sexual dynamics; fulfillment of the female role; changing perceptions of breasts; and various coping practices (Table 2).

**Table 2:** A summary of themes, subthemes, and key codes

Main Themes	Subthemes	Key codes
Avoidance of sexuality	Physical changes in sexual dynamics	• Vaginal dryness and painful sexual intercourse • Breast milk leakage during sexual activity • Engorgement and tingling sensations • Discomfort related to episiotomy following vaginal birth • Pain or sensitivity at the cesarean section incision site • Insomnia and fatigue associated with frequent breastfeeding
	Psychological changes in sexual dynamics	• Decreased sexual desire • Feelings of disgust when breasts are touched • Embarrassment caused by breast milk leakage • Time pressure, fear of waking the infant, and rushed sexual intercourse • Difficulty achieving orgasm and reduced sexual pleasure • Fear of unintended pregnancy while breastfeeding • Feelings of guilt related to thoughts of the baby during sexual intercourse due to the dominance of the maternal role
	Religious, social, and cultural changes in sexual dynamics	• Religion-based disapproval of sexual activity during breastfeeding • Avoidance of sexual intercourse due to the presence of family members (e.g., mother or mother-in-law) in the household • Reluctance to breastfeed without performing ritual ablution (ghusl) • Perception of sexuality as impure during the breastfeeding period • Feelings of being observed due to sharing the same room with the infant
Maintaining sexuality as a responsibility	Fulfillment of the female role	• Maintaining sexual intercourse primarily to satisfy the partner • Acceptance of sexual intercourse despite pain or discomfort to fulfill perceived female role expectations
	Changing roles of breasts	• Positive effects of breast enlargement on sexual body image • Transformation of breasts from sexual organs to instruments of infant nourishment
Coping with the effects of breastfeeding on sexuality	Various coping practices	• Passive coping through waiting, with the belief that difficulties will resolve over time • Negotiating sexual boundaries with the partner (e.g., avoiding breast contact) • Wearing a bra during sexual intercourse to prevent milk leakage • Desire for healthcare professionals to educate men about breastfeeding-related sexual challenges

### Theme 1: Avoidance of Sexuality

Participants reported exhibiting avoidance behaviors related to sexual activity during the postpartum period. This avoidance was attributed to a combination of physical, psychological, religious, social, and cultural changes experienced after childbirth.

Accordingly, the theme Avoidance of Sexuality comprised three subthemes: physical changes in sexual dynamics; psychological changes in sexual dynamics; and religious, social, and cultural changes in sexual dynamics.

#### Physical Changes in Sexual Dynamics

Women indicated that their sexual lives were adversely affected by physical complications following childbirth, particularly discomfort associated with episiotomy and cesarean section incisions. Although physical complications following childbirth are not the primary focus of this study, they are presented first in accordance with the chronological flow of participants’ experiences. This ordering reflects how early postpartum physical changes constitute the initial context within which breastfeeding and sexuality are subsequently experienced and negotiated.

I had a vaginal birth and stitches. It felt as difficult as my first sexual experience. Fear, excitement, desire, and obligation were all mixed together. Even though the doctor said the stitches would dissolve and heal on their own, I was afraid they would open. I tensed up so much that my husband had to stop the intercourse; my legs were shaking. (P1)

I had a cesarean section, and my stitches still hurt. We have tried different positions. The areas where the stitches were placed, especially the edges, still cause discomfort. Sometimes my husband has to stop intercourse, and when he is on top, I feel a stinging sensation in my abdomen. It has been almost six months, but I still experience difficulty during intercourse. (P3)

Vaginal dryness that developed during breastfeeding led to painful intercourse, while breast fullness, tingling sensations, and milk leakage disrupted intimacy and reduced sexual pleasure.

I experienced very severe vaginal dryness. Once, I even had to use Vaseline. It hurt so much that after intercourse, I felt sensitivity for two days, even while urinating. Whenever my husband wants to have sex, I become scared and tense my legs because I expect pain. Because of this, my husband said he did not enjoy it either. (P4)

I experienced milk leakage from my breasts while breastfeeding. When they are full, there is a strong tingling sensation, and it hurts immediately if my husband tries to touch them. That is why I do not want to have sex. (P2)

#### Psychological Changes in Sexual Dynamics

Participants reported that the physical effects of breastfeeding were closely intertwined with psychological changes that negatively influenced their sexual experiences. In particular, vaginal dryness and pain during intercourse often resulted in avoidance of sexual activity.

I avoid intercourse because it hurts and causes pain. I think my husband also has concerns, so he does not want to have sex either. We try occasionally without forcing the issue, but the fear of pain is very strong. (P7)

Women also described developing sexual aversion due to persistent fatigue, sleep deprivation, fear of the baby waking during intercourse, and anxiety about becoming pregnant while breastfeeding.

I don’t want to have sex because I’m tired and sleepless. Even if my husband wants to, we worry that the baby will wake up, so I say, “Never mind, let’s not do it.” (P3)

I don’t want to get pregnant again. My aunt’s daughter became pregnant while breastfeeding, and there is only a 13-month gap between her children. The last pregnancy resulted in twins, and she is devastated. I’m afraid the same thing could happen to me. (P5)

Women stated that they could not enjoy sex or orgasm due to the time pressure created by rushed sexual intercourse out of fear of waking the child.

I would describe it as rushed sex. I can’t focus on pleasing my husband because I’m afraid the baby will wake up; there’s no foreplay. Naturally, this doesn’t give me pleasure. We’re just relieved if it ends before the baby wakes. (P10)

I don’t really want sex; there’s a lack of desire. Even if we have intercourse, I can’t orgasm. We have to wait until the baby falls asleep. Before having a child, we had sex two or three times a week; now we’re grateful if it’s twice a month. Men can meet their needs, but what about women? Being a woman is really hard, you’re responsible for the child and for the husband, but no one asks what you want or how you cope. (P4)

Feelings of disgust and shame were also commonly reported, particularly when breasts were touched or when breast milk leaked during intercourse. These experiences were often accompanied by guilt associated with the dominant motherhood role.

Maybe he sees me like a cow to be milked. I remember the first time we tried to have sex; he told me, mockingly, that I smelled of milk while kissing me. I laughed it off, but it hurt. If anyone smells like milk, it’s our daughter. Why should I smell like milk? But he continued anyway. (P1)

There is leakage and tingling in the breasts, and milk comes out. It’s a complete source of disgust. Once, my husband didn’t want to have intercourse because of the milk. He said, ‘Let’s not do it.’ (P2)

At first, he thought the wetness on his arm was the baby’s urine, but it was my milk. It had all leaked out. I was extremely embarrassed. I felt like a cow, not a woman. When the milk leaks, I think of my baby and feel guilty because the milk that should be feeding my baby is being wasted. (P8)

#### Religious, Social, and Cultural Changes in Sexual Dynamics

Participants emphasized that their attitudes toward sexuality during the breastfeeding period were strongly shaped by the religious, social, and cultural values of their communities. Women stated that they did not view sexuality during breastfeeding favorably, they perceived it as impure, and that they did not breastfeed their babies without performing a full ablution (ghusl).

It is necessary to perform a full ablution after sexual intercourse. I don’t want to breastfeed my baby without doing so. Sometimes I avoid intercourse just because of this. If the baby wakes during intercourse, I rush to the bathroom to take a shower and perform ablution, while the baby cries in the father’s arms. Breastfeeding without ablution, especially without a full ablution, is not appropriate. I want my baby to receive clean milk. (P9)

It becomes a problem because of having to perform a full ablution. We were taught that you shouldn’t even sit at the breakfast table without washing your hands and face as soon as you wake up. How could I breastfeed my own child without performing ablution after sexual intercourse? Having to rush to perform ablution afterward keeps me away from intercourse. (P4)

Some women reported that being in the same room with the baby during breastfeeding made them feel like someone was watching them during intimacy, and that they could not have sex when there were people in the same house supporting the baby’s care, such as the mother and mother-in-law.

The baby’s crib is in our bedroom, so before intercourse, we take the baby to the living room or another room, or we move ourselves. We can’t say, ‘It’s just a baby; it won’t understand.’ It feels like someone is watching us, and that makes us feel ashamed. We have never had sex in front of our baby. (P3)

After giving birth, my mother-in-law stayed with us. During the day, intimacy was not possible, and at night it was also impossible. Sometimes she would take care of the baby so I could rest, but I couldn’t even hug my husband because she might come into the room at any moment. You have to respect elders. Being intimate with your spouse when there are elders in the house feels inappropriate and embarrassing. If she heard us, she might think we were having sex. (P2)

### Theme 2: Maintaining Sexuality as a Responsibility

Participants described maintaining sexual activity during the breastfeeding period primarily as a perceived obligation associated with being a partner. Many women reported engaging in sexual intercourse to fulfill what they described as their “duties” or responsibilities toward their partners, even in the absence of desire or sexual satisfaction. Participants also noted that breastfeeding altered their perceptions of their bodies, particularly their breasts, and that maternal identity became more dominant during this period. Accordingly, the theme Maintaining Sexuality as a Responsibility comprised two subthemes: fulfillment of the female role and changing roles of breasts. Although the changing role of breasts may appear primarily physiological or psychological, participants often framed this transformation within the context of relational expectations and perceived responsibilities to maintain sexual intimacy. Therefore, this subtheme is situated under “maintaining sexuality as a responsibility” to reflect how these changes were experienced and interpreted in relation to partner dynamics and perceived obligations.

#### Fulfilling the Female Role

The findings revealed that women frequently perceived sexuality as a functional requirement of partnership and womanhood. As a result, sexual intercourse was often maintained despite a lack of desire or pleasure, primarily to satisfy partners and fulfill perceived gendered responsibilities.

Instead of focusing on sex, my mind is always on the baby. I feel like I could hear the baby’s voice at any moment. I don’t experience sexual pleasure at all. One ear is always listening for the children, especially the baby. If the baby wakes up, the other child will wake up too. My motherhood outweighs everything; my femininity comes second. If I didn’t have responsibilities toward my husband, I wouldn’t force myself to have sex. I’m forcing myself for him. (P2)

As I said, he can satisfy himself. But I feel responsible toward him. I feel that I can’t fulfill my role as a woman. After all, isn’t it a woman’s duty to satisfy her husband? (P5)

#### Changing Roles of Breasts

Participants reported that breastfeeding substantially altered their perceptions of their breasts. Many women stated that breasts lost their sexual connotations and were instead viewed primarily as organs belonging to the infant and as instruments of nourishment. Consequently, breast contact during sexual intercourse was often perceived as uncomfortable or inappropriate. They also stated that they shared this with their partners before starting sexual intercourse and reached a mutual agreement not to touch their breasts.

Breastfeeding is a wonderful feeling. Seeing my baby’s pleasure gives me pleasure too. But when my husband touches my breasts, that doesn’t give me pleasure at all. That’s why we agreed from the beginning that the breasts would not be touched. (P2)

The baby can’t suckle from both breasts at the same time. When we’re about to start intercourse, my husband sometimes laughs because he can tell which breast the baby fed from, one is full, the other is saggy and deflated. I don’t know whether to laugh or cry. I tell my husband that I am a mother before anything else. That’s why I tell him from the beginning, “You won’t touch the breasts; they’re not yours.” (P4)

In contrast, some participants reported that physical changes associated with breastfeeding, particularly breast enlargement, had a positive effect on their body image and their partners’ sexual perceptions.

The fact that my breasts have grown makes me happy physically. My husband is happy too; he says there’s something more to touch. I hope my breasts stay this way even after I stop breastfeeding. (P3)

My breasts have grown because of breastfeeding, and my husband even said that he likes this. The appearance of my breasts gives him pleasure. In that sense, breastfeeding has had a positive effect on my husband. (P6)

### Theme 3: Coping with the Effects of Breastfeeding on Sexuality

Participants described developing various individual strategies to cope with the effects of breastfeeding on their sexual lives or adopting a passive approach based on the belief that these difficulties would resolve over time. Accordingly, this theme comprised the subtheme of various coping practices.

#### Various Coping Practices

Many women expressed the belief that sexual difficulties experienced during the breastfeeding period were temporary and would diminish naturally over time.

I’m not doing anything in particular; I just tell myself that this process will pass. I experienced these problems with my previous births as well, and now I’m experiencing them again. They pass with time. (P1)

Women used various methods to cope with the problems they experienced with sexuality during breastfeeding. They mentioned practices such as setting boundaries with partners by asking them not to touch their breasts during intercourse, not removing their bra to reduce the effect of milk leakage, and expressing milk before starting intercourse. Furthermore, women emphasized the need for health professionals to provide education for men regarding problems experienced during breastfeeding.

I don’t take off my bra so that the milk won’t make me disgusted. (P2)

Actually, what happens to wives should also be explained to men so that they too know what goes on during this process. (P3)

First, I make an agreement that he won’t touch my breasts. If he doesn’t touch them, it’s not much of a problem. Also, in the evening, I express the breast that the baby hasn’t suckled. I even bought a breast pump for that. (P4)

## Discussion

This study investigated the perceptions and experiences of breastfeeding mothers between the third and sixth months postpartum regarding breast milk and sexuality. The findings were discussed within the framework of three main themes: avoidance of sexuality, maintaining sexuality as a responsibility, and coping with the effects of breastfeeding on sexuality.

### Avoidance of Sexuality

Sexuality changes at different stages of a woman’s life for various reasons, and the postpartum period is one of the stages during which female sexuality is particularly affected (O’Malley et al., 2021). The literature suggests that breastfeeding affects sexual function through hormonal and physiological changes, particularly increased prolactin levels and decreased estrogen levels. These changes may result in vaginal dryness, dyspareunia, and decreased libido, thereby negatively affecting sexual life. Accordingly, the frequency of sexual dysfunction has been reported to be higher among breastfeeding women (Alp Yılmaz et al., 2019; Sun et al., 2024). Consistent with the literature, our findings showed that women tended to avoid sexual intercourse due to physiological changes associated with breastfeeding, such as vaginal dryness, dyspareunia, breast engorgement, and milk leakage, as well as insomnia and fatigue related to frequent breastfeeding. In addition, obstetric factors related to childbirth, such as pain, tenderness, and prolonged healing following episiotomy or cesarean section, were found to negatively affect women’s ability to resume and maintain sexual activity. Previous studies reporting associations between postpartum sexual problems and assisted vaginal delivery, cesarean delivery, and exclusive breastfeeding support our findings (Matthies et al., 2019; O’Malley et al., 2018). Similarly, a qualitative study conducted in Spain revealed that women did not feel ready to initiate or sustain sexual life after childbirth and demonstrated avoidance behaviors (Pardell-Dominguez et al., 2021). These findings suggest that biological changes in the postpartum period, particularly those related to lactation, directly influence sexual function through physiological discomforts, contributing to the avoidance of sexuality.

Although uterine contractions caused by the natural release of oxytocin during breastfeeding are known to create sensations similar to orgasm in some women (Yurtsal & Uslu, 2023), hormonal changes associated with breastfeeding may also lead to increased breast tenderness, milk leakage during intercourse, decreased vaginal lubrication, vaginal atrophy, reduced sexual desire, difficulties with arousal and pleasure, feelings of sexual unattractiveness, and anxiety related to a potential new pregnancy (Serrano Drozdowskyj et al., 2020). In our study, women reported avoiding sexual activity during breastfeeding due to psychological and emotional factors, including decreased sexual desire, feelings of disgust when their breasts were touched, embarrassment related to milk leakage, time pressure, fear of waking the baby, rushed sexual encounters, inability to achieve orgasm, reduced sexual enjoyment, fear of pregnancy while breastfeeding, and the intrusion of the maternal role, such as thinking about their baby, during intercourse.

Previous research has shown that the dual role of breasts during breastfeeding may cause anxiety among women, with many feeling discomfort when their breasts are used as a source of sexual pleasure by their partners while simultaneously serving as a means of feeding their infants (O’Malley et al., 2019). Similarly, another study reported that sexual relationships were negatively affected by psychological, sociocultural, and infant-related factors, despite women’s expectations that their sexual satisfaction would eventually improve (Kul Uçtu & Karakoç, 2022). Consistent with the existing literature, our findings demonstrate that breastfeeding influences sexuality not only through physiological changes but also through complex psychological processes.

Although sexuality is a universal aspect of human life, it is shaped and interpreted differently depending on the social, cultural, and religious context in which individuals live (Rahmani et al., 2021). In our study, women reported avoiding sexuality during breastfeeding for religious, social, and cultural reasons. These included perceiving sexuality unfavorably during breastfeeding, being unable to engage in sexual activity in the presence of caregivers such as mothers or mothers-in-law in the household, the belief that breastfeeding without performing ritual ablution (ghusl) was inappropriate, perceiving sexuality as impure during breastfeeding, and feeling as though they were being watched when sharing the same room with their baby. Supporting these findings, one study indicated that 22.6% of women believed sexual intercourse should be prohibited during breastfeeding, while 13% thought that sexual activity could harm the baby during this period (Bader et al., 2024). Another study showed that the physical and daily life changes experienced during breastfeeding were often attributed negative meanings, which in turn adversely affected women’s sexual lives (Queiroz et al., 2024). Together, these findings highlight the importance of understanding breastfeeding not only as a physiological process but also as a socially and culturally embedded experience that significantly shapes women’s perceptions of sexuality.

### Maintaining Sexuality as a Responsibility

When breastfeeding is examined from a psychobiological perspective, it is emphasized that varying degrees of conflict may arise between a woman’s sexuality and her maternal role during this period (Perlman, 2019; Queiroz et al., 2024). In our study, women reported engaging in sexual intercourse during breastfeeding primarily to satisfy their partners, which they perceived as a responsibility associated with their female or spousal role. Despite experiencing pain and discomfort, participants described accepting sexual intercourse to meet their partners’ sexual needs.

In traditional societies, sociocultural norms, values, and beliefs strongly influence women’s infant-feeding practices and their attitudes toward continuing sexual activity during the postpartum breastfeeding period (Smetanina et al., 2025). The findings of our study are consistent with the existing literature and suggest that multiple factors experienced during breastfeeding, particularly religious and cultural norms that frame sexuality as conflicting with breastfeeding, may contribute to challenges in maintaining both roles. Such conflicts may also play a role in the early cessation of breastfeeding. Supporting this interpretation, a study conducted in Israel showed higher breastfeeding rates and longer breastfeeding durations among Christian and Druze mothers compared with Muslim mothers (Awawdi et al., 2024). Therefore, especially in societies that have adopted Islam, the religious and cultural dimensions of sexuality that cause women to stop breastfeeding early need to be examined in the postpartum period.

Although women in our study frequently reported avoiding sexuality during breastfeeding, some participants stated that they continued sexual activity because breast enlargement associated with lactation positively influenced their sexual body image. In this context, breasts were perceived not only as a means of nourishment but also as contributing to sexual attractiveness. While some studies have shown that larger breast size and increased nipple stimulation during breastfeeding may positively affect sexuality, enhance sexual satisfaction, and facilitate orgasm (Anbaran et al., 2015; Grussu et al., 2021), other research has reported contrasting findings. For example, Bender et al. (2018) found that women often did not perceive themselves as sexually attractive due to changes in breast size and instead viewed their breasts primarily as belonging to their infants, making it difficult to associate them with sexuality. Overall, while our findings are largely consistent with the literature, it appears that women’s self-perceptions vary during breastfeeding, and they may react differently to sexuality while breastfeeding. In this context, studies focusing on factors that cause differences in experience between breastfeeding and sexuality, such as partner reaction, religiosity, sexual education, and body image, are needed.

### Coping with the Effects of Breastfeeding on Sexuality

In our study, women adopted various strategies to cope with the effects of breastfeeding on their sexuality. Some participants reported taking no specific action, believing that the difficulties they experienced would resolve naturally over time. Others described actively modifying their sexual practices, such as not allowing their partners to touch their breasts before or during sexual intercourse, expressing breast milk in advance to prevent leakage, or keeping their bras on during sexual activity. In addition, women emphasized the importance of health professionals informing male partners about the physical and emotional challenges associated with breastfeeding.

The literature suggests that sexuality during breastfeeding extends beyond penetrative intercourse and that sexual difficulties during this period should be approached as temporary and self-limiting. It has been recommended that couples focus on mutual communication, emotional closeness, affection, erotic thoughts, and non-penetrative forms of intimacy, including touching and physical closeness, to maintain their sexual relationship during this time (Undiyaundeye, 2016). Although one study explored coping strategies related to the physical, emotional, and lifestyle changes associated with postpartum sexual problems, it did not specifically address the impact of breastfeeding on sexuality or the coping strategies used by breastfeeding women (Delgado-Pérez et al., 2022). The coping strategies identified in this study provide important insight into how breastfeeding women navigate changes in their sexual lives during the postpartum period. Although the qualitative design does not allow for direct comparisons in terms of sexual satisfaction between women who do and do not use specific coping strategies, the findings suggest that these strategies may play a significant role in how women interpret and manage sexual difficulties.

Women who reported using coping strategies appeared to actively engage in adaptive processes such as redefining sexual intimacy, adjusting expectations regarding sexual activity, and prioritizing emotional closeness over physical intimacy during the postpartum period. These adaptive mechanisms may contribute to a more flexible interpretation of sexual satisfaction, even in the presence of physical or psychosocial barriers. In contrast, women who did not report using such strategies may experience postpartum sexual changes as more disruptive, potentially perceiving these changes as more distressing or problematic. This suggests that coping strategies may function as a protective factor in maintaining relational and sexual well-being, although this interpretation should be approached cautiously given the exploratory nature of the study. Overall, these findings highlight the clinical relevance of supporting women in developing adaptive coping mechanisms during the postpartum period, particularly in contexts where cultural expectations and physical recovery processes may intersect to influence sexual experiences.

In this context, the findings of our study are particularly valuable, as they provide insight into the coping practices women use to manage breastfeeding-related sexual challenges. While postpartum follow-ups, home visits, digital platforms, telephone counseling, and breastfeeding applications are commonly used to support the early initiation and continuation of breastfeeding, sexual education and counseling provided by health professionals to couples remain crucial. Such interventions can facilitate communication and intimacy between partners, help balance social and family roles, and ultimately promote sexual health during the breastfeeding period (Hajimirzaie et al., 2021).

### Limitations and Strengths

Research examining the effects of breastfeeding on sexuality among breastfeeding women in Türkiye remains limited. Therefore, this study makes a valuable contribution to the literature by presenting breastfeeding women’s perceptions and lived experiences related to sexuality, which constitutes a key strength of the study.

However, several limitations should be acknowledged. First, the study was conducted in a single center, and only Turkish-speaking women were included. Additionally, women’s perceptions and experiences may vary across different regions and sociocultural contexts. For this reason, future studies conducted in diverse geographical and cultural settings are warranted. Another limitation of this study is that participants included women with different modes of delivery, such as cesarean section and vaginal delivery with suturing, which may have influenced postpartum sexual experiences due to factors such as pain and physical recovery. These differences may have affected participants’ engagement in sexual activity independent of breastfeeding. Therefore, the findings should be interpreted with consideration of the potential influence of delivery-related factors. Finally, as this study employed a qualitative research design with a relatively small sample size, the findings cannot be generalized to all women.

### Conclusion

The findings of this qualitative study, which explored postpartum women’s perceptions and experiences of breastfeeding and sexuality, indicate that breastfeeding and sexuality are interrelated through physical, psychological, social, cultural, and religious factors. During the postpartum period, beyond the physiological effects of breastfeeding on sexual function, women’s interpretations and meanings attributed to breastfeeding and sexuality play a central role in shaping their sexual experiences. These interpretations may lead to avoidance of sexuality or to behaviors in which sexuality is perceived as a responsibility or duty.

Women experience conflicts and dilemmas between breastfeeding, their maternal role, and their spousal role, and they employ various strategies to cope with these challenges. Based on these findings, it is recommended that women be monitored during the postpartum period with regard to the effects of breastfeeding on sexuality. Additionally, couples should be informed about the potential impacts of breastfeeding on sexual health and effective coping strategies. Integrating the topic of sexuality during breastfeeding into prenatal education programs and planning further studies that examine breastfeeding women’s perceptions and experiences across different cultural contexts are also recommended.

## Data Availability

Not applicable.

## Appendix: Semi-Structured Interview Form

1. Do you think your sexuality has been generally affected after childbirth? If so, could you describe how?
2. Do you think your partner’s sexuality has been generally affected after childbirth? Could you elaborate on this?
3. How would you describe your feelings or emotions regarding the possible effects of breastfeeding on your sexuality during the postpartum period?
4. In what ways, if any, has breastfeeding affected your sexuality?
5. In what ways, if any, has breastfeeding affected your partner’s sexuality?
6. Some hormones released during breastfeeding may have physical effects on women’s sexuality. How do you think you and your partner have been physically affected during this period?
7. Some hormones released during breastfeeding may have psychological effects on women’s sexuality. How do you think you and your partner have been psychologically affected during this period?
8. From social, cultural, or religious perspectives, how has engaging in sexual activity during the breastfeeding period affected you and your partner?
9. Some couples report difficulties in maintaining sexual intimacy during the postpartum period due to issues such as milk leakage or concerns about the baby waking during intercourse. Have you or your partner experienced similar situations, and if so, how have they affected your sexual relationship?
10. What strategies or practices have you used to cope with the negative effects of breastfeeding on your sexual life, if any?
